# Feed in summer, rest in winter: microbial carbon utilization in forest topsoil

**DOI:** 10.1186/s40168-017-0340-0

**Published:** 2017-09-18

**Authors:** Lucia Žifčáková, Tomáš Větrovský, Vincent Lombard, Bernard Henrissat, Adina Howe, Petr Baldrian

**Affiliations:** 1 Institute of Microbiology of the CAS, Vídeňská 1083, 14220 Praha 4, Czech Republic; 2 Faculty of Science, Charles University, Albertov 6, 128 43 Praha 2, Czech Republic; 3 Architecture et Fonction des Macromolécules Biologiques, CNRS, Aix-Marseille Université, Marseille, France; 4 INRA, USC 1408 AFMB, Marseille, France; 5 Department of Biological Sciences, King Abdulaziz University, Jeddah, Saudi Arabia; 6 Iowa State University, Ames, IA USA

**Keywords:** Auxiliary activity enzymes, Bacteria, Carbon cycle, Carbohydrate-active enzymes, Coniferous forests, Decomposition, Fungi, Glycoside hydrolases, Lignocellulose-degradation, Seasonality, Transcriptomics

## Abstract

**Background:**

Evergreen coniferous forests contain high stocks of organic matter. Significant carbon transformations occur in litter and soil of these ecosystems, making them important for the global carbon cycle. Due to seasonal allocation of photosynthates to roots, carbon availability changes seasonally in the topsoil. The aim of this paper was to describe the seasonal differences in C source utilization and the involvement of various members of soil microbiome in this process.

**Results:**

Here, we show that microorganisms in topsoil encode a diverse set of carbohydrate-active enzymes, including glycoside hydrolases and auxiliary enzymes. While the transcription of genes encoding enzymes degrading reserve compounds, such as starch or trehalose, was high in soil in winter, summer was characterized by high transcription of ligninolytic and cellulolytic enzymes produced mainly by fungi. Fungi strongly dominated the transcription in litter and an equal contribution of bacteria and fungi was found in soil. The turnover of fungal biomass appeared to be faster in summer than in winter, due to high activity of enzymes targeting its degradation, indicating fast growth in both litter and soil. In each enzyme family, hundreds to thousands of genes were typically transcribed simultaneously.

**Conclusions:**

Seasonal differences in the transcription of glycoside hydrolases and auxiliary enzyme genes are more pronounced in soil than in litter. Our results suggest that mainly fungi are involved in decomposition of recalcitrant biopolymers in summer, while bacteria replace them in this role in winter. Transcripts of genes encoding enzymes targeting plant biomass biopolymers, reserve compounds and fungal cell walls were especially abundant in the coniferous forest topsoil.

**Electronic supplementary material:**

The online version of this article (10.1186/s40168-017-0340-0) contains supplementary material, which is available to authorized users.

## Background

Coniferous forests in the boreal and temperate zones of the Northern Hemisphere represent significant global carbon pools and sinks [1]. Consequently, understanding the processes of the carbon (C) cycle and its changes during the year is essential for modeling potential impacts of global climate change on these ecosystems. Forest ecosystems are influenced by trees that mediate the influx of C into the ecosystem pool and contribute to soil respiration and carbon deposition, which are important microbial-mediated processes [2–4], although the relative share of fungi and bacteria and effect of the seasonality on these processes, remains largely undiscovered.

In the coniferous forest floor, litter and soil represent largely different compartments when considering C cycling processes [5, 6]. The litter is derived from recalcitrant plant biopolymers (cellulose, hemicelluloses, and lignin). In contrast, soil is a mixture of highly recalcitrant materials, such as polyphenols—products of lignin degradation, and has a low abundance of plant biopolymers. The tree roots exude photosynthesis-derived labile C compounds that enter soil both directly and through root-associated ectomycorrhizal fungi (ECM) [3, 5]. Another important C pool is found in living biomass of microbiota (chitin and peptidoglycan) or in storage compounds (starch, glycogen, and trehalose) [7–9].

The difference in the content of C matter between horizons of forest floor leads to the microbial community stratification [5, 7, 10]. Litter is rich in saprotrophic taxa, while ECM fungi, mostly represented by Basidiomycota in our ecosystem [5], tend to dominate the soil [11]. The relative abundance of bacteria increases with depth, and the composition of their communities differs in litter and soil as well [5].

The turnover of C compounds can be tracked by the analysis of enzymes that mediate them—the carbohydrate-active enzymes (CAZymes). Specifically, glycoside hydrolases (GH) and selected auxiliary activity enzymes (AA) are associated with the decomposition of polysaccharides and lignin, respectively [12]. The classification of GH and AA proteins or genes into families that contain structurally similar proteins makes it possible to assign catalytic functions to sequences using CAZy pipeline [12].

CAZymes have previously been studied in forest soil transcriptomes, though only by approaches targeting individual genes by PCR [5, 13] or exclusively eukaryotic transcripts at low throughput exploiting Sanger sequencing [14]. While previous studies of litter proteomes have indicated the dominance of fungal decomposition enzymes over bacterial ones [15], the results of stable isotope probing experiments indicate that fungi and bacteria are both involved significantly in cellulose and hemicellulose utilization in forest soils [16–19]. On the other hand, bacteria dominated in the utilization of dead fungal biomass, which is an important pool of C in forest topsoil [20]. The results of aforementioned studies indicate that fungi and bacteria are involved in decomposition of different substrates, but the share of fungi and bacteria in degradation of these substrates is still unknown.

Seasonality in temperate forest soils is composed of individual and well documented factors controlling soil microbial communities [21–27], such as a change in soil moisture, soil temperature, and plant activity that strongly affects C cycling in forest ecosystems [28]. One of the approaches to study the effect of seasonality on C cycling is to measure activities of microbial CAZymes in the studied environment. It was found that activities of CAZymes depend on temperature as the main driving factor of seasonal differences in enzymatic activities in the tundra [29]. Temperature may cause decline of CAZymes activities in the cold season compared to the activities in the warm season in both boreal coniferous forest [30] and in temperate spruce forest [31], and thus also negatively affect C cycling. Changes in the C cycling by microbial community and in the composition of microbial community were observed between summer and winter in mixed temperate forest [32] as well as in an arctic system [33]. Dominance of saprotrophic fungi, which are largely responsible for degradation of lignocellulose [34], in spring and ECM fungi, which are involved in plant derived C storage in soil [3], in late summer was shown for the temperate and boreal forests [35–39]. In case of ECM fungi, it is thought to be supported by maximal growth of spruce fine roots in the summer, with which they are symbiotic [40]. Bacterial community composition also responded to the seasonal changes in temperate forest [10, 41] and alpine soils [28, 42], and it was interpreted as a reaction to C fluctuations in plant roots exudates during the year as well as with variation in moisture and temperature of soil.

The photosynthetic activity of trees during the summer period is high (with favorable temperature and light conditions), while it is minimal in winter, when it is reduced by light limitations and temperatures below the freezing point. Consequently, carbon allocation belowground varies dramatically and directly impacts soil biota [4, 43]. In our previous study, we have demonstrated that the presence of microbial communities in the coniferous *Picea abies* forest topsoil is similar among seasons, but their activities differ dramatically [44]. The pool of transcripts differs among seasons, especially in the soil, where fungal transcripts were observed to significantly decrease (by 50%) in winter, with ECM-associated activity being particularly reduced [44].

The consequences of seasonality on the C cycle in soil are not well known, but it can be hypothesized that the reduced input of photosynthates in the form of root exudates in winter, and continuous demand of microorganisms for C sources would be seen as an increase in the transcription of genes encoding CAZymes for the utilization of recalcitrant plant C compounds, such as cellulose and hemicellulose by saprotrophic bacteria and fungi. To survive the winter season, we expect ECM fungi to subsist on plants storage compounds, such as starch, or on their own storage compounds, such as glycogen and trehalose, and to transcribe the genes for relevant CAZymes, such as amylases and trehalases, respectively.

In this study, for the first time, we describe the contribution of fungi and bacteria to CAZyme pool in two different seasons, summer and winter, leveraging the power of metagenomics and metatranscriptomics with sufficient reliability and resolution. We use metagenomics to characterize functional potential of individual taxa for CAZyme production, and metatranscriptomics to describe the contribution of microorganisms to CAZyme share between seasons and so mediated effect on C-cycling.

## Methods

### Sampling area and sample collection

The study sites were located in the highest altitudes of the Bohemian Forest National Park, Czech Republic (49° 2′ 38″ N, 13° 37′ 2″ E), covered by an unmanaged Norway spruce (*P. abies*) forest. The mean annual temperature was 5 °C, and the mean annual precipitation was 1000 mm. The soil temperatures at the days of sampling were recorded and can be find in the study of [44], but the soil moisture was not recorded. When the understory was present, it was composed of grasses (*Avenella*, *Calamagrostis*), bilberries (*Vaccinium*), mosses, and ferns. This study used the samples of DNA and RNA collected in July 2012 and March 2013 previously described in the study of [44]. Briefly, six samples were taken from the litter layer (L) and the organic horizon of soil (S). After removal of roots, L material was cut into 0.5 cm pieces and was mixed, while S material was passed through a 5-mm sterile mesh and was mixed. A total of 24 samples were collected (six sites × two seasons × two horizons). Samples were immediately frozen in liquid nitrogen and stored at − 80 °C until analysis.

### Extraction and analysis of environmental RNA and DNA

For all 24 samples, RNA and DNA extraction, the metatranscriptome sequencing and assembly were described previously by [44]. Briefly, RNA was extracted using the RNA PowerSoil Total RNA Isolation Kit (MoBio Laboratories) combined with the OneStep PCR Inhibitor Removal Kit (ZymoResearch) from three 1-g aliquots per sample, pooled, and RNA-purified using the RNA Clean & Concentrator kit (ZymoResearch) on a column treated with DNase I (Fermentas). Approximately 1 μg of RNA was treated with an equimolar mixture of RiboZero rRNA Removal Kits Human-Mouse-Rat and Bacteria (Epicenter) and a total of 50 ng of treated RNA served as the input for the ScriptSeq v2 RNA-Seq Library Preparation Kit (Epicenter) that was used for library construction. Total DNA was extracted in triplicate from all samples as showed in [44]. DNA samples were fragmented to reach the mean fragment lengths around 400 bp and libraries were prepared using TruSeq PCR Free Kit (Illumina). Metatranscriptome and metagenome libraries were sequenced on an Illumina HiSeq2000 to generate 150-base paired-end reads.

Metagenome reads were processed in the same way as originally described for the metatranscriptome [44]. The reads were quality trimmed by removing adapters with Trimmomatic (v 0.27) using Illumina TruSeq2-PE adapters with a seed mismatch threshold, palindrome clip threshold, and simple clip threshold set at 2, 30, and 10, respectively [45]. Furthermore, sequencing reads were filtered by base call quality using the FASTX-Toolkit (http://hannonlab.cshl.edu/fastx_toolkit/index.html), specifically fastq_quality_filter, with the following parameters: -Q33 -q 30 -p 50. Resulting sequences were normalized using methods previously described in [46, 47] and Khmer (v 0.7.1) and command normalize-by-median.py with the following parameters: -k 20 -C 20 -N 4 -x 50e9. Next, errors were trimmed by removing low abundance fragments of high coverage reads with Khmer and command filter-abund.py -V. The paired-end assembly of the remaining reads was performed with the Velvet assembler (v 1.2.10, -exp_cov auto -cov_cutoff auto -scaffolding on [48]) using odd k-mer lengths ranging from 33 to 63. Resulting assembled contigs were merged using CD-HIT v4.6 [49, 50] and minimus2 Amos v3.1.0 [51]. Assembly of metagenomic reads was performed in the same way as described for the metatranscriptome and sequence data of all contig sequences have been deposited in the MG RAST public database [52] under the dataset number 4627252.3; metatranscriptome contigs are available as 4544233.3. Metagenome sequencing yielded 567 × 10^6^ reads (24 × 10^6^ ± 2 × 10^6^ reads per sample) that were assembled into 9,380,309 contigs over 200 bases, including 1,882,204 contigs over 500 bases, 569,720 over 1000 bases and 6665 over 10,000 bases (mean length was 454 bases). The longest contig had a length of 179,090 bases. Protein prediction in MG-RAST yielded a total of 9,178,489 predicted coding regions, of which 4,355,554 (47.5%) have been assigned an annotation by MG RAST.

### Annotation of the metagenome and metatranscriptome

Contig annotation was first performed in MG RAST with an E value threshold of 10^−5^ while also considering the representative hit option (i.e., single best annotation for each feature) and taxonomic information was retrieved for each identified contig. Because MG RAST is not suitable for annotation of fungal proteins, predicted proteins were subsequently annotated by finding the best protein match in an in-house database containing protein predictions from all publicly available fungal genomes available at the time of analysis (155 genomes). For all hits that received closer hit in terms of E value to the fungal-predicted protein database (FPPD) then using MG RAST, taxonomic information was retrieved from the FPPD.

Glycoside hydrolases (GH) and auxiliary enzymes (AA) were identified among the metagenome and metatranscriptome contigs using the CAZy pipeline [12], which combines Blast and HMM tools with the manual curation of CAZy database (http://www.CAZy.org). Protein models are compared with the sequence and profile libraries created from catalytic and non-catalytic modules of the CAZy database. GH and AA families were grouped based on which substrate they act upon, with the guidance of Dr. Bernard Henrissat, the founder of CAZy database (Table 1). We considered AA families to be involved in degradation of lignin because lignin is always found in union with polysaccharides in the plant cell wall and thus AA fill a variety of enzyme mechanisms to act on substrates related to lignocellulose conversion [53].

**Table 1:** Functional classification of glycosyl hydrolases and auxilliary used in this paper based on their characterized catalytic activities according to CAZy (http://www.CAZy.org)

Group	Target	GH families
Cellobiose	Cellooligosaccharides	GH1 (β-glucosidase/β-xylosidase), GH3 (β-glucosidase/β-xylosidase/endoxylanase), GH116 (β-glucosidase/β-xylosidase)
Cellulose	Plant cell wall	GH5_1, GH5_2, GH5_4, GH5_5, GH5_25, GH5_26, GH5_38, GH5_39, GH5_46 (endocellulase), GH6 (exocellulase/endocellulase), GH7 (exocellulase/endocellulase), GH8 (endocellulase/endoxylanase), GH9 (exocellulase/endocellulase/endoxylanase/β-glucosidase), GH12 (endocellulase/endoxylanase), GH44 (endocellulase/endoxylanase), GH45 (endocellulase), GH48 (exocellulase/endocellulase/chitinase), GH74 (endocellulase), AA9 (lytic polysaccharide monooxygenase)
Chitin	Glucans fungal cell wall	GH5_9, GH5_14, GH5_15 (β-1,3-glucanase/ β-1,6-glucanase), GH16 (endo-1,3-β-glucanase/endo-1,3-β-galactanase), GH17 (endo-1,3-β-glucosidase/ β-1,3-β-glucosidase), GH18 (chitinase), GH19 (chitinase), GH20 (β-hexosaminidase), GH72 (β-1,3-glucanosyltransglycosylase)
Lignin	Plant cell wall	AA1 (laccase), AA2 (peroxidase), AA3 (oxidase), AA4 (oxidase), AA5 (oxidase), AA6 (oxidase), AA12 (oxidase)
Pectin	Plant cell wall	GH5_7, GH5_8, GH5_10, GH5_17, GH5_19, GH5_36 (β-mannanase/endo-β-1,4-mannanase), GH28 (polygalacturonase/rhamnogalacturonase), GH62 (α-L-arabinofuranosidase), GH78 (α-Lrhamnosidase), GH88 (β-glucuronyl hydrolase), GH105 (glucuronyl hydrolase/galacturonyl hydrolase), GH106 (α-L-rhamnosidase)
Peptidoglycan	Bacterial cell wall	GH22 (lysozyme), GH23 (lysozyme), GH24 (lysozyme), GH25 (lysozyme), GH108 (lysozyme)
Starch/glycogen	Storage compounds	GH13 (amylase/α-glucosidase/trehalase), GH14 (amylase), GH15 (glucoamylase/glucodextranase), GH31 (α-glucosidase), GH57 (amylase), GH77 (amylomaltase), GH119 (amylase)
Trehalose	Storage compounds	GH37 (trehalase), GH65 (trehalase)
Xylan	Plant cell wall	GH5_22 (β-xylosidase), GH10 (endo-1,4-β-xylanase/endo-1,3-β-xylanase), GH11 (endoxylanase), GH30 (endoxylanase/β-xylosidase/β-glucosidase), GH67 (xylan α-1,2-glucuronidase), GH115 (xylan α-1,2-glucuronidase), GH120 (β-xylosidase)

To assess the abundance in the metagenome and the relative rate of transcription in the metatranscriptome, individual sequence reads from each sample were mapped onto contigs identified as GH or AA using bowtie 2.2.1 [54] with the default settings of end to end alignment—sensitive. To calculate gene or transcript abundance, data were expressed as: per base coverage = read count × read length/contig length. Abundances were always reported as normalized values, i.e., shares of all transcripts in given sample, or, where indicated, shares of all transcripts of a selected microbial taxon.

### Statistical analysis

R [55] and PAST 3.03 (http://folk.uio.no/ohammer/past/) were used for statistical analysis. Differences in relative abundances of gene or transcript abundances were tested using the Mann-Whitney *U* test with the Bonferroni correction for multiple comparisons. The Mann-Whitney *U* assumes the measurements on a rank-order scale but does not assume normality of data. One-way or two-way PERMANOVA on Bray-Curtis distances with 99,999 permutations was used to analyze differences among communities or transcript pools. Two-dimensional non-metric multidimensional scaling (NMDS) ordination analysis on Bray-Curtis distances was performed in R with package vegan [55, 56]. In all cases, differences at *P* < 0.05 were considered to be statistically significant.

## Results

### Transcription of genes encoding auxiliary enzymes and glycoside hydrolases

**Fig. 1:**
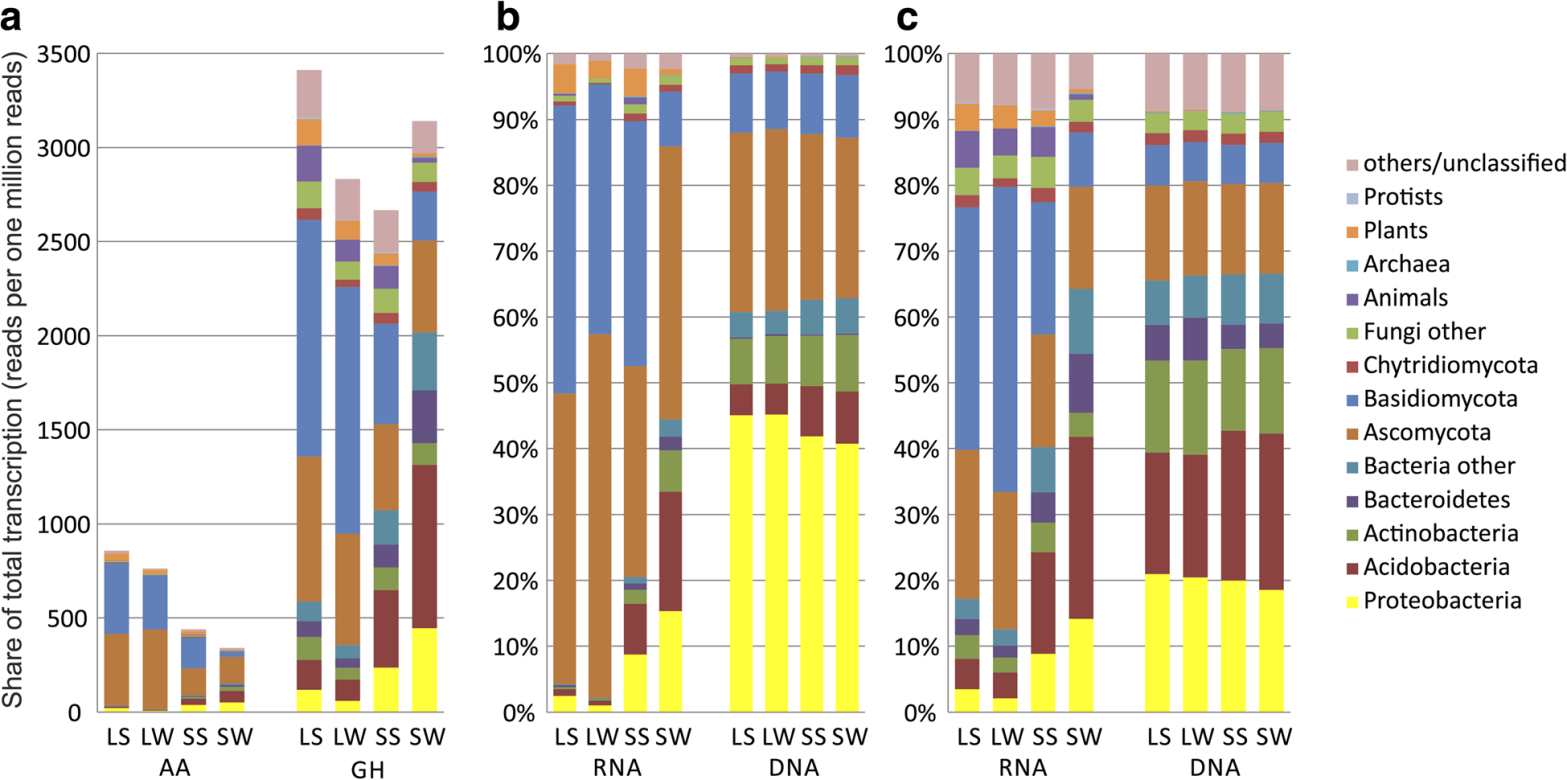
Contribution of taxa to the transcription and gene pool in the *P. abies* topsoil. **a** The share of AA and GH reads in the total metatranscriptome in ppm (reads per one million reads). **b** The share of organisms on the transcription of glycoside hydrolases. **c** Auxilliary enzymes in percentage. Abbreviations: *LS* litter summer, *LW* litter winter, *SS* soil summer, *SW* soil winter

Thirteen percent of 4.5 million of all contigs from *P. abies* topsoil metatranscriptome were assigned to carbohydrate metabolism [44], while the rest of transcripts belonged mainly to housekeeping genes and other metabolic pathways [44]. 42,872 (0.83%) transcripts were identified as GH belonging to 105 families and 5111 (0.11%) as AA belonging to 12 families. The transcription of GH represented between 0.26 and 0.34% of the total transcription in both litter and soil. AA were more transcribed in litter (0.07–0.08% of total transcription) than in soil (0.03–0.04%; Fig. 1a). GH13 (amylase) was the most diverse family with 4574 contigs followed by GH5 (mixed activities on cellulose, hemicellulose and pectin) (2011 contigs), GH3 (β-glucosidase) (1707 contigs). High diversity of transcription was found in families AA3 (mixed activities on lignocellulose) (1683 contigs) and AA1 (laccase) that had more than 1000 contigs (Additional file 1: Table S1).

**Fig. 2:**
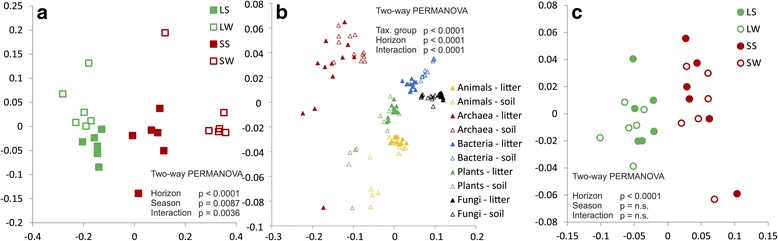
Nonmetric multidimensional scaling of the of GH and AA in the *P. abies* topsoil. **a** Relative abundances of all CAZymes in metatranscriptome by horizons and seasons. **b** The composition of the pools of GH and AA transcribed by higher taxa of organisms in litter and soil. **c** Relative abundances of all CAZymes in gene pools by horizons and seasons. Abbreviations: *LS* litter summer, *LW* litter winter, *SS* soil summer, *SW* soil winter

**Fig. 3:**
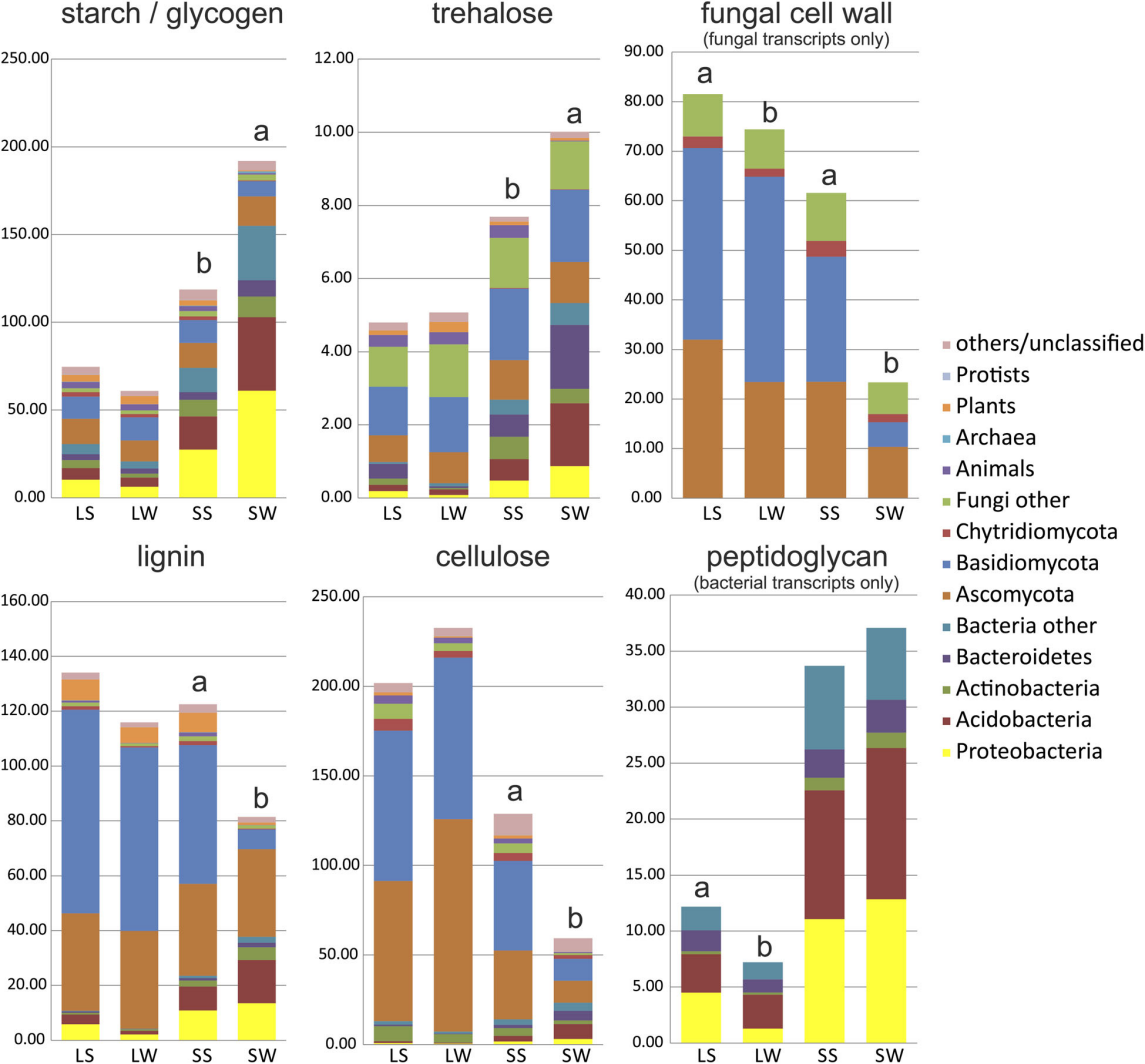
Transcription of GH and AA by functional groups in *P. abies* forest topsoil by seasons. Numbers indicate the share of reads in the total metatranscriptome in ppm (reads per one million reads). Significant differences in read abundances among seasons are indicated by different letters. Abbreviations: *LS* litter summer, *LW* litter winter, *SS* soil summer, *SW* soil winter

**Fig. 4:**
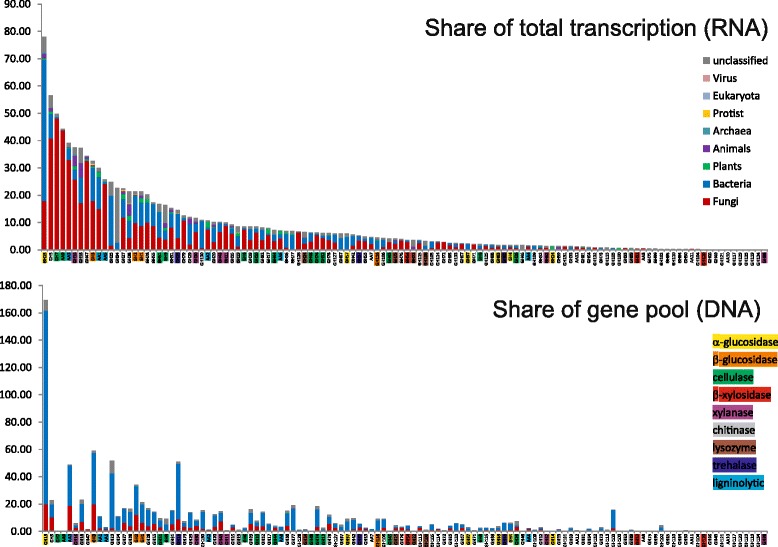
Share of AA and GH families with abundance over five unit of ppm on total transcription and gene pool in the *P. abies* topsoil. Read abundances are in ppm (reads per one million reads). Colors of stack bars indicate taxonomic affiliation of transcripts and genes, functional groups of CAZymes are color-coded in name of the CAZy family

GH and AA transcripts were more frequently of fungal origin (43% GH and 71% AA) then of bacterial origin (42 and 22%). Most GH and AA transcripts were transcribed by fungi that accounted for 51.6% of GH reads (27.6% Basidiomycota, 19.3% Ascomycota) and as much as 81.5% of AA reads (44.7% Ascomycota, 35.1% Basidiomycota). Bacteria were responsible for 34.7% of GH transcription (Acidobacteria 13.7%, Proteobacteria 7.6%), and 13.1% of AA transcription. GH were also frequently transcribed by animals (3.7%) and plants (2.6%) and AA by plants (3.3%; Fig. 1b, c). It was apparent that groups of organisms are significantly different in enzyme sets they produce (Fig. 2b). All taxa transcribe α-glucosidases and cellulases, however, fungi had the highest share on transcription of cellulases and ligninolytic enzymes. All species also transcribed β-glucosidases and chitinases, while archaea did not transcribe xylanases and only some Proteobacteria transcribe xylanases (Additional file 1: Table S2). In plants, the most transcribed genes belonged to AA2 (class II peroxidase), AA1 (laccase), GH27 (α-galactosidase), and GH38 (mannosidase). The contribution of individual groups to the production of CAZymes was highly variable with a high contribution of fungi to the production of the dominant plant cell wall-degrading enzymes—cellulases, ligninases (up to 90% of fungal transcripts in both groups), and xylanases (up to 70%); all other enzyme groups were produced by a wide range of taxa (Figs. 3 and 4).

Among the functional groups of CAZymes as defined in Table 1, the ones targeting cellulose were most transcribed followed by those acting on lignin, fungal cell wall, and starch and glycogen. Most metatranscriptome reads were associated with the α-glucosidases of the GH13 family, with the cellulolytic enzymes in families GH5 (subfamilies 1, 2, 4, 5, 25, 26, 38, 39, 46), GH7 and AA9, ligninolytic oxidases AA3, endoglucanase/endogalactanase GH16, and with the chitinase GH18 (Fig. 4). Among cellulolytic genes, transcripts from AA9 family were very abundant in litter (27% in summer and 40% in winter), while transcripts of GH7 family were mostly produced in summer in both horizons (36%).

The transcript pool differed significantly between litter and soil: 86 gene families showed significantly different transcription rate between horizons (Additional file 1: Table S1), which probably reflects differences in microbial community composition [5, 44] as well as differences of C sources between horizons [6]. Soil samples showed higher transcription of CAZymes targeting reserve compounds (starch/glycogen and trehalose) and peptidoglycan, while the genes encoding enzymes degrading cellulose and lignin and pectin were more transcribed in litter (Fig. 3), which suggests different source utilization between horizons, with plant-derived polymers to be more important C source in litter, while dead biomass of bacteria and root exudates in soil.

### Seasonality of transcription

The transcript pools differed significantly between summer and winter in both horizons but seasonality was more pronounced in soil, where it affected 60 CAZy gene families compared to 18 families in litter (Fig. 2a, Additional file 1: Table S1). At the level of functional groups of CAZymes, differences in their metagenome content among seasons was negligible as well as the variation in the taxonomic groups that produced them (Additional file 2: Figure S1). Most of seasonal differences in transcription among the functional groups of CAZymes as classified in Table 1 and the shift in the contribution of taxa to their production (Fig. 3) was found especially in soil.

Winter samples were marked by an increase in the use of reserve compounds (glycogen/starch and trehalose) while the share of CAZymes targeting recalcitrant plant biomass (cellulose and lignin) decreased. Significantly higher transcription of CAZymes targeting fungal cell wall components, such as chitin, and selected glucans was observed in summer compared to winter (Fig. 3), indicating higher turnover and growth rates in the warm season. Transcription of CAZymes involved in bacterial peptidoglycan degradation in litter was also higher in summer than in winter. For all enzyme groups, the share of CAZymes transcribed by fungi in soil decreased in winter while the contribution of bacteria increased (Fig. 3). For example, fungi transcribe 62% of cellulases in soil in summer but only 29% in winter.

Of 2836 CAZyme-associated transcripts that appeared in at least five litter samples, 219 (7.7%) were significantly increased in summer and 103 (3.6%) in winter. In soil, of 2119 transcripts, 287 (13.5%) were increased in summer and 215 (10.1%) in winter confirming more dramatic change in distribution of transcripts in soil than in litter. In soil, CAZymes significantly more transcribed in summer were those targeting cellulose, lignin and microbial cell walls, while CAZymes targeting starch, glycogen, and trehalose were more frequently transcribed in winter (Fig. 3, Additional file 3: Figure S2).

### Gene pool of auxiliary enzymes and glycoside hydrolases

Among genes predicted in soil metagenomes, 5.5% of genes were annotated as CAZymes and the annotations for the rest of gene families can be found in study of [44]. In total, 91,195 GH from 108 families and 7709 AA from 11 families were identified among the protein predictions of the *P. abies* topsoil metagenome. GH13 was the most diverse family with 16,412 contigs followed by GH3 and GH15 (trehalase) and another 29 GH families and the family AA3 were identified in > 1000 contigs (Fig. 4, Additional file 1: Table S1). Most abundant families in metagenome were GH13, GH3, GH23 (chitinase), GH15, AA3, GH2 (β-glucosidase), GH18 (chitinase), GH5 (cellulolytic subfamilies 1, 2, 4, 5, 25, 26, 38, 39, 46), and GH1 (β-glucosidase) (Fig. 4) in total representing approximately one half of all reads.

Among contigs identified as GH, 67% were of assigned to bacteria and 27% to fungi and of AA 46% were of bacterial and 24% of fungal origin. Considering the frequency of occurrence, 66.2% of glycoside hydrolase reads were assigned to bacteria (Proteobacteria 20.0%, Acidobacteria 20.9%, Actinobacteria 13.4%, Bacteroidetes 4.9%) and 24.9% to fungi (Ascomycota 14.1%, Basidiomycota 6.1%) (Fig. 1 b). Of auxiliary enzyme reads, 61.8% were assigned to bacteria (Proteobacteria 43.2%, Acidobacteria 6.3%, Actinobacteria 7.6%) and 37.5% to fungi (Ascomycota 26.1%, Basidiomycota 9.1%). Reads assigned to other organisms were rare (Fig. 1c).

The gene pool differed significantly between litter and soil, but not between seasons (Fig. 2c). Relative abundance of 120 individual gene families was significantly different among horizons for 72 gene families but among season only for 9 gene families in litter and 2 gene families in soils, indicating that the community composition is horizon-specific but similar in both seasons (Additional file 1: Table S1).

## Discussion

### Gene pool of CAZymes and its transcriptions

Our results confirmed that all CAZy families were transcribed by many taxa at the same time and more isoforms of CAZymes were found in the genomes of the most abundant species of bacteria binned from metagenome (data not shown), which suggest high functional redundancy in soil environment, confirming the observations previously made for another functional gene cellobiohydrolase (cbhI), which was also produced simultaneously by hundreds of taxa [5].

The share of fungi in our metatranscriptome is two times higher than bacteria, while in the metatranscriptomic studies from a maple forest or in peatlands, bacterial CAZy transcripts were 2.6 to 5-fold more abundant then eukaryotic ones [57, 58]. This may indicate high importance of fungi in coniferous forests, although the comparison may be biased by the fact that previous studies annotated short reads and used a limited reference database for fungi, so that relevant transcripts might have been overlooked. The observed dominance of fungal-associated CAZymes is consistent with previous results obtained by proteomic analysis of decomposing beech litter, where fungal transcripts also dominated the enzyme pool [15]. Compared to that study, we have observed a much higher share of transcripts from Basidiomycota, which is in agreement with their high abundance in the studied ecosystem [44].

Our data confirm that the share of bacterial reads in the metagenome is higher than in the metatranscriptome, while the opposite is true for fungal genes and transcripts. The low representation of eukaryotic genes that is generally reported from microbial metagenomic studies [59, 60] may be caused by the high abundance of non-coding DNA in eukaryotic sequences, which may be overcome by using eukaryotic metatranscriptomes that contain mostly coding sequences. Also, eukaryotic transcription is unrelated to the amount of gene copies in genomes because of its complex regulation [61]. Another cause of low eukaryotic abundance in metagenomes can be the underrepresentation of fungal and other eukaryotic genomes in genomic databases [62] and thus the inability to properly identify genes of these organisms.

CAZymes in both the metagenome and metatranscriptome, differed significantly in their composition between litter and soil. This is consistent with the fact that the composition of bacterial and fungal communities differs among horizons in the studied ecosystem [5] as well as elsewhere [10, 63], reflecting the properties of these habitats, such as the availability of C sources [64]. Litter was enriched in transcripts associated with cellulases and lignin-targeting enzymes, indicating the importance of decomposition of recalcitrant plant biopolymers. Soil showed an increased share of enzymes acting on glycogen and especially trehalose, which both are known reserve compounds of fungi, including ECM fungi [65].

### Involvement of soil organisms in C transformation

The vast majority of GH and AA (83–92% and 93–95%, respectively) were encoded and transcribed by microorganisms, i.e. fungi, bacteria but the share of archaeal reads in metatranscriptome and metagenome was negligible. Fungi accounted for the higher share of AA transcription then bacteria, and this was also the case of GH transcription in litter; in soil, GH were produced equally by fungi and bacteria in summer, but bacteria largely dominated GH transcription in soil in winter.

The share of bacterial transcription of GH and AA was substantial in soil, and bacterial transcripts were associated with the majority of all CAZymes, the most abundant bacterial CAZymes were the ones involved in degradation of labile substrates, such as starch, cellobiose or other oligosaccharides. Concretely, bacterial GH13 genes, which are putative α-glucosidases/α-amylases, were the most abundant in metagenomes and in metatranscriptomes as well. GH13 genes, were among the most abundant CAZymes genes present in bacterial genomes [66] and were also found to be abundant in another forest soil [59] and highly transcribed in peatlands [58]. Bacterial transcripts of CAZymes with chitinolytic activity confirmed important role of bacteria in the turnover of dead fungal mycelia [20].

Acidobacteria, Proteobacteria, Bacteroidetes, and Actinobacteria were the most abundant bacterial producers of CAZymes. These groups are known to be abundant in acidic forest topsoil [44] and have also been found to be dominant in CAZy production in another acidic environment—the boreal peatland [58]. The culturing and analysis of Acidobacteria, Proteobacteria, and Bacteroidetes from coniferous forests has confirmed the production of a wide range of extracellular enzymes by the individual members of these phyla, especially by the Acidobacteria and Bacteroidetes [67]. On the one hand, it was also showed that bacteria are able to produce a CAZymes that allow them to access C in cellulose or hemicelluloses [16, 19, 68]. On the other hand, the decomposition of lignin, cellulose was dominated by fungi that appear to be better adapted to decompose recalcitrant plant-derived biomass components [69]. Fungal CAZymes that were associated with plant biomass decomposition were identified in dominant transcripts of both Ascomycota and Basidiomycota and were related to cellulolytic enzymes, namely, the GH7 cellulases and the AA9 lytic polysaccharide monooxygenases. Overall, the fungi are the major producers of CAZymes involved in lignocellulose degradation, while bacteria highly transcribed CAZymes involved in the degradation of storage compounds and bacterial and fungal cell walls. In other groups of organisms, for example plants, mannosidases of family GH38 and laccases of AA1 belonged to the most transcribed CAZymes. GH38 are necessary for the correct root development [70] and laccases perform many roles, such as lignin polymerization [71] and tissue repair [72].

### Seasonality of C utilization

Here, we confirm the previous observation that composition of microbial communities in litter and soil in both seasons remains stable as observed elsewhere [73–75], but our results from the same ecosystem indicate that the ratio of fungi to bacteria and fungal biomass are significantly higher in summer than in winter [44]. We also show that transcripts of genes encoding enzymes for fungal and bacterial cell wall degradation are higher in summer than in winter, which may indicate higher growth rates in summer for fungi and bacteria, respectively. The seasonality of transcription of GH and AA was substantially higher in soil as it was also the case for total transcription in the studied ecosystem [44] and likely reflects the differences in seasonal fluctuations of C sources, of which tree photosynthates are highly important, being responsible for 30% of soil microbial respiration in coniferous forest soils [4].

The relative contribution to CAZyme transcription increased in soil in winter for presumed saprotrophs such as the Ascomycota and bacteria. This observation may either indicate the relief of inhibition of non-mycorrhizal microorganisms due to removal of ECM competitors, so called the Gadgil effect [76] or simply the decrease of activity of ECM fungi, which is more likely [44]. The observation of the increase in transcription of CAZymes associated with mobilizing reserve compounds—starch, glycogen, and trehalose in winter soil indicates that this is likely a time of nutrient limitation, and biomass may need to be maintained at the costs of metabolic reserves. Starch from plant roots [77] and glycogen from bacteria/fungi [78] was utilized mainly by bacteria in soil during the winter. Bacteria were found to be the main starch consumers in SIP experiment [79] as well. The use of trehalose and mannitol as energy reserves by ECM during winter starvation has been observed in previous studies [65]. The observed decrease in the transcription of GH and AA by Basidiomycota in winter in soil is consistent with the decrease of transcription of genes related to ECM symbiosis [44], indicating lower activity of the ECM fungi, mainly Basidiomycetes [5].

Contrary to our expectations, the fungal communities in soil did not switch from the utilization of labile C compounds in summer to more recalcitrant carbohydrates in winter. Instead, the transcription of genes involved in lignin, cellulose, and xylan degradation was increased in the summer and the transcription of genes degrading simple C compounds such as α- and β-glucosidases and amylases was similar in winter and summer. However, we hypothesize that in winter ECM fungi utilize α-glucosidases and amylases to survive on storage compounds, while in summer they thrive on root exudates, degradation of which, fuel the production of ligninolytic enzymes in search of ECM fungi for N in recalcitrant OM [63]. It was demonstrated previously that summer microbial community decomposes labile C sources, such as root exudates or glucose, rapidly [80–82], and there is experimental evidence that labile substrates may prime decomposition of recalcitrant OM [83–85]. An alternative explanation, such as that seasonal differences in transcription of CAZymes are driven by temperature is less plausible, because it logically suggests the lower activity of both fungi and bacteria in winter but the decrease of trnascription was specific only for ECM fungi [44]. Although we still lack an understanding of the differences in metabolic resting states in fungi and bacteria, the most plausible solution to the problem of decreased transcription in winter in ECM fungi seems to be diminished supply of C from the symbiotic trees. Although we did not follow the seasonal changes in DOC and SOC composition, our results support the hypothesis that C priming may be necessary for the decomposition of complex biopolymers by fungi.

## Conclusions

Organisms in coniferous litter and soil possess a diverse set of enzymes that participate in decomposition of complex C compounds. Microorganisms are the most important producers of these enzymes, especially GH and AA CAZymes, with fungi strongly dominating transcription in litter and equal contributions of bacteria and fungi in soil. Composition of microbial community, as far as phyla representations, remains stable across the year, but gene transcription shows seasonality in terms of different abundance of CAZymes transcripts assigned to different microorganisms and their share. Our results indicate that transcription of CAZymes involved in fungal biomass turnover is higher in summer than in winter, while the use of reserve compounds such as starch or trehalose is high in winter. Seasonality of gene transcription is especially high in soil where summer is characterized by high transcription of ligninolytic and cellulolytic CAZymes. Although, both fungi and bacteria contribute to CAZy transcription, our results confirmed the leading role of fungi in the degradation processes as confirmed by the fact that thy produced more than half the observed CAZymes.

## Additional files

Additional file 1: Supplementary Tables. (XLSX 53 kb)

Additional file 2: Supplementary Figure 1. (PDF 1453 kb)

Additional file 3: Supplementary Figure 2. (PDF 1423 kb)

## Abbreviations

AA: Auxiliary carbohydrate-active enzymes,
C: Carbon
CAZy: Carbohydrate active enzymes
ECM: Ectomycorrhizal fungi
FPPD: Fungal-predicted protein database
GH: Glycoside hydrolases
N: Nitrogen
OM: Organic matter
