# Supplementary material for: Feed in summer, rest in winter: microbial carbon utilization in forest topsoil
Source: Microbiome. 2017 Sep 18;5:122. doi: 10.1186/s40168-017-0340-0 (PMC5604414; doi:10.1186/s40168-017-0340-0)
Supplement: Supplementary file 2 — Supplementary Figure 1. (PDF 1453 kb) [file 40168_2017_340_MOESM2_ESM.pdf]

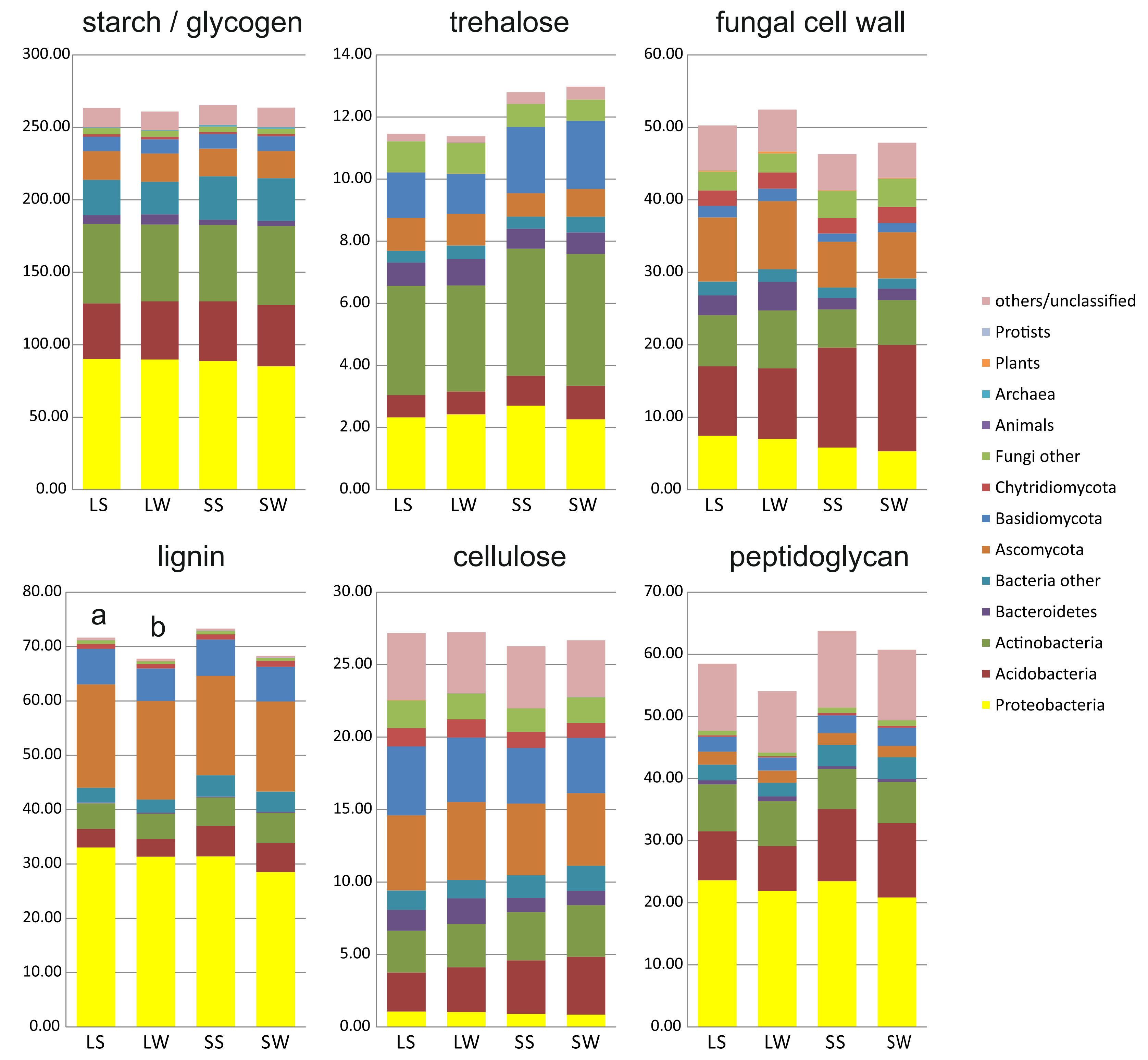

Figure S1.: Read abundances of GH and AA by functional groups in the metagenome of litter and soil of the *Picea abies* forest. Numbers indicate the share of reads in the total metatranscriptome in ppm. Significant differences in read abundances among seasons are indicated by different letters. LS - litter summer, LW - litter winter, SS - soil summer, SW - soil winter.
