# Supplementary material for: Feed in summer, rest in winter: microbial carbon utilization in forest topsoil
Source: Microbiome. 2017 Sep 18;5:122. doi: 10.1186/s40168-017-0340-0 (PMC5604414; doi:10.1186/s40168-017-0340-0)
Supplement: Supplementary file 3 — Supplementary Figure 2. (PDF 1423 kb) [file 40168_2017_340_MOESM3_ESM.pdf]

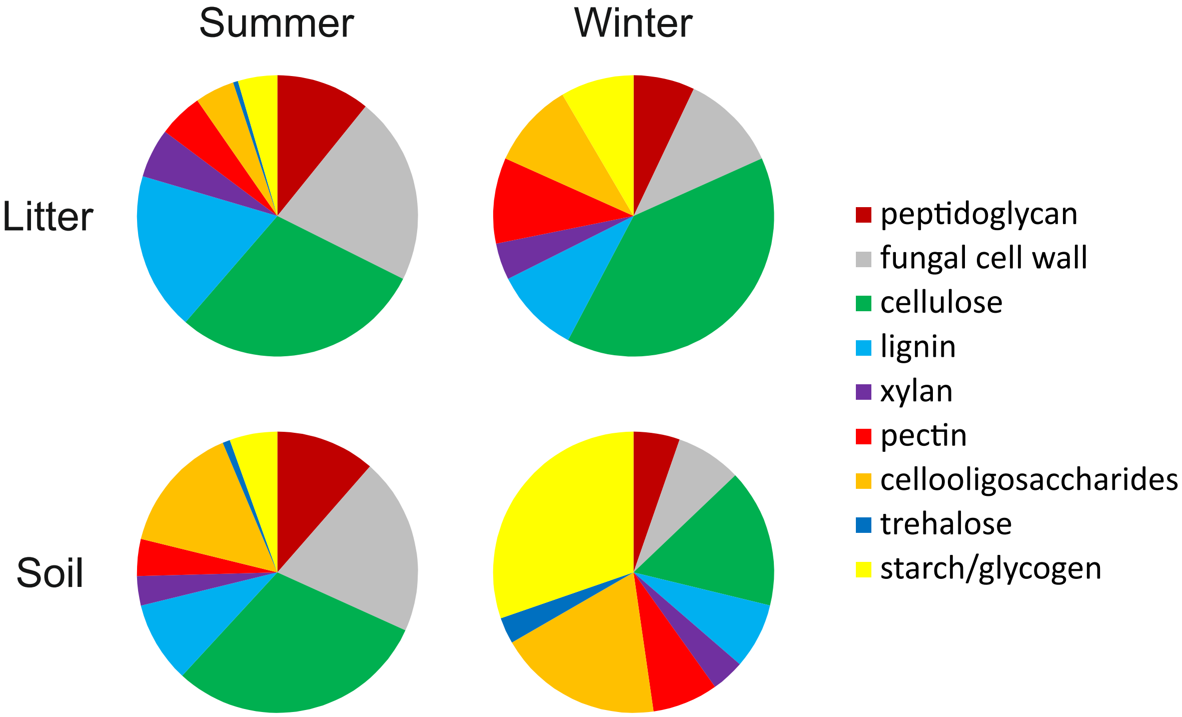

Figure S2.: Functional classification of GH and AA genes with significantly higher expression in summer and in winter within litter and within soil of a *Picea abies* forest.
